# Some *CYP21A2* Polymorphisms in the Exon 7 Region Might Be Associated with Cortisol Secretion in Polycystic Ovary Syndrome

**DOI:** 10.3390/jcm15124626

**Published:** 2026-06-14

**Authors:** Ralitsa Robeva, Silvia Andonova, Georgi Kirilov, Iglika Yordanova, Silvia Vandeva, Atanaska Elenkova, Alexey Savov, Tihomir Todorov

**Affiliations:** 1 Department of Endocrinology, Faculty of Medicine, Medical University-Sofia, USHATE “Acad. Iv. Penchev”, 1000 Sofia, Bulgaria; drgkirilov@abv.bg (G.K.); svandeva@yahoo.com (S.V.); aelenkova@medfac.mu-sofia.bg (A.E.); 2 Genetic Medico-Diagnostic Laboratory “Genica”, 1000 Sofia, Bulgaria; sandonova@netscape.net (S.A.); yordanova.iglika@gmail.com (I.Y.); tisho.todorov@abv.bg (T.T.); 3 National Genetic Laboratory, Medical Faculty, Medical University-Sofia, University Hospital of Obstetrics and Gynecology “Maichin Dom”, 1000 Sofia, Bulgaria; alexey.savov@abv.bg

**Keywords:** PCOS, non-classic CAH, genetic polymorphisms, *CYP21A2*

## Abstract

**Background**: Polycystic ovarian syndrome (PCOS) and the non-classic form of congenital adrenal hyperplasia (NC-CAH) are hyperandrogenic conditions with overlapping clinical symptoms but different genetic backgrounds. The possible interrelationships between the two conditions remain unclear; thus, the present study aims to investigate the prevalence of *CYP21A2* exon 7 genetic variants in patients with PCOS and to explore the possible associations of the polymorphisms with adrenocortical hormonal production. **Methods**: The *CYP21A2* exon 7 region was genotyped in 80 unrelated female patients with PCOS and 12 women with NC-CAH. The associations between genetic variants, clinical characteristics, and adrenocortical hormones were investigated. **Results**: The pathogenic *CYP21A2* NC-CAH variant c.844G>T; p.(Val282Leu) was found in 66.7% (8/12) of patients with NC-CAH but in none of the individuals with PCOS. The benign rs1554305325, rs6465, rs6472, and rs6477 genetic polymorphisms were not related to clinical hyperandrogenism. The rs6472 polymorphic alleles were associated with increased adrenocorticotropic hormone (ACTH) (5.5 vs. 3.4 pmol/L, *p =* 0.022) and cortisol (460.5 vs. 366.5 nmol/L, *p =* 0.016) levels. The rs6465 variant alleles were significantly associated with lower pregnenolone (1.43 vs. 3.1 ng/mL, *p =* 0.031) and ACTH (2.5 vs. 4.5 pmol/L, *p =* 0.030) levels in the unadjusted model but not after adjustment for potential confounders (*p* > 0.05). **Conclusions**: The *p*.(Val282Leu) variant is very common among Bulgarian patients with NC-CAH but it has not been found in our cohort of women with PCOS. The *CYP21A2* exon 7 polymorphisms might be associated with cortisol levels in the patients with PCOS. Further larger studies are needed to confirm or reject the current findings in different ethnic groups.

## 1. Introduction

Polycystic ovary syndrome (PCOS) is a heterogeneous condition affecting 1 in 10 women and is characterized by metabolic, reproductive, and psychological disturbances [1,2]. Androgen excess is among the most common complaints in the affected patients, but the exclusion of other diseases associated with hyperandrogenism is mandatory for the proper PCOS diagnosis [3,4]. For instance, non-classic congenital adrenal hyperplasia (NC-CAH) with late presentation might be clinically indistinguishable from PCOS in adolescent and adult females [5]. Increased adrenal androgens and 17-OH-progesterone have been found in both disorders despite different genetic backgrounds and various disturbances in steroidogenic pathways [6]. NC-CAH is an autosomal recessive disease caused mainly by a mild-to-moderate decrease in 21-hydroxylase enzyme activity leading to exaggerated adrenal androgen synthesis [7]. On the other hand, PCOS is a polygenic-determined condition associated with 17-hydroxylase/17,20-lyase overexpression and, therefore, enhanced ovarian and adrenal androgen production in most women [8]. Cortisol secretion is usually retained or slightly decreased in women with NC-CAH [7]. Conversely, in patients with PCOS, it might be normal or exaggerated because of an abnormal stress response or relative hypoglycemia in insulin-resistant individuals [9,10].

Alterations in adrenal steroidogenesis may be crucial for the development of PCOS symptoms; thus, it is prudent to evaluate possible associations between variations of the 21-hydroxylase gene (*CYP21A2*) and clinical and hormonal characteristics of patients with PCOS. According to Huang et al., patients with PCOS with hirsutism exhibited a significantly higher carrier rate of NC-CAH variants compared with non-hirsute women [11]. Additionally, *CYP21A2* intron 2 polymorphisms have been associated with adrenal androgen excess in PCOS [12]. The most common pathogenic variant found in patients with NC-CAH is *CYP21A2*(NM_000500.9): c.844G>T, p.Val282Leu (*CYP21A2*:c.841G>T/p.V281L, according to older nomenclature), but its prevalence might differ significantly in distinct ethnic groups [13,14]. Therefore, the present study aims to investigate the prevalence of this genetic variant in patients with PCOS of Bulgarian (Caucasian) origin and to explore the possible associations between polymorphisms in the *CYP21A2* exon 7 region and clinical and hormonal characteristics of PCOS.

## 2. Materials and Methods

### 2.1. Patients and Study Protocol

A total of 80 unrelated female patients (age 18–45 years) with PCOS diagnosed based on the Rotterdam criteria [3,4] were investigated. Additionally, 12 unrelated women (age 18–47 years) with NC-CAH, proven hormonally, were evaluated as positive controls. NC-CAH was diagnosed based on morning basal or Synacthen-stimulated 17-OH progesterone levels ≥ 30 nmol/L (<300 nmol/L) per the current guideline [7].

The patients with PCOS were selected consecutively from the women consulted at a tertiary endocrine clinic. The presence of hirsutism (Ferriman–Galway score ≥ 8), acne, polycystic ovaries, and menstrual disturbances was evaluated and recorded based on current guidelines [4]. The other possible causes for clinical complaints, e.g., CAH, Cushing syndrome, prolactinoma, premature ovarian failure, androgen-producing tumors, and overt (non-treated) hypothyroidism were excluded by appropriate tests. Patients with severe concomitant diseases that might influence hormonal levels were also excluded from the study. None of the patients were treated with corticosteroids; two had received oral contraceptives in the last three months, and 28 (35%) of the patients were on metformin therapy. Autoimmune thyroid disease was established in 20 patients (25%), with 12 of them (15%) being on L-thyroxin treatment.

Anthropometric (height, weight, body mass index [BMI]), biochemical, and hormonal investigations were performed in all patients. Blood samples were collected at 08–09:00 a.m. after a 12 h overnight fast in the early follicular phase of the menstrual cycle or in amenorrhea. Biochemical investigations included measurements of fasting glucose, high-density lipoprotein cholesterol (HDL-ch), low-density lipoprotein cholesterol (LDL-ch), triglycerides (TG), and total cholesterol. The biochemical parameters were measured enzymatically by an automatic analyzer (Roche Cobas E411). The hormonal investigations included total testosterone, dehydroepiandrosterone sulfate (DHEAS), and adrenocorticotropic hormone (ACTH), determined by electrochemiluminescence methods (Roche Cobas E411). Blood samples for ACTH were specifically collected in pre-chilled EDTA plasma tubes, transported on ice, and centrifuged at 4 °C with subsequent immediate plasma separation and measurement. The intra-assay coefficients of variation (CVs) were below 3.2%, while inter-assay CVs were below 5.3% for all parameters. Additionally, 17-OH progesterone and cortisol were determined by commercially available radioimmunological kits, with intra-assay CVs below 4.6% and inter-assay CVs below 7.6%. Pregnenolone levels were determined by ELISA, with intra-assay CV 10.6% and inter-assay CV 14.5% (Diagnostics Biochem Canada Inc., London, ON, Canada).

Blood samples with K_2_EDTA were obtained from all patients with PCOS and CAH and used for genetic analysis of the *CYP21A2* exon 7 region, including the intron–exon boundaries. All patients provided written informed consent for participation in this study. This study was approved by the Ethics Committee of the Medical University, Sofia (Protocol №15/11 June 2025).

### 2.2. Genetic Analysis

The standard salt extraction method was used for obtaining genomic DNA from all participants. The genetic analysis for patients with PCOS and NC-CAH was targeted on the *CYP21A2* exon 7 region and determined the pathologic variant c.844G>T; p.(Val282Leu). All samples were successfully genotyped. In women carrying the pathogenic c.844G>T; p.(Val282Leu) variant according to the initial analysis (*n* = 8), all *CYP21A2* gene coding regions and exon–intron boundaries were additionally evaluated by Sanger sequencing and MLPA to confirm the findings. The test was performed using highly specific primers according to the in-house-modified protocol, as previously described [12]. The regions of interest were Sanger-sequenced with specific internal primers and BigDyeTerminator Cycle Sequencing Kit, v.3.1 (Thermo Fisher Scientific, Waltham, MA, USA) in accordance with the manufacturer’s instructions. After electrophoresis on the ABI3130xl Genetic DNA Analyzer (Thermo Fisher Scientific, Waltham, MA, USA), electropherograms were visualized using SeqScape software v2.7 (Thermo Fisher Scientific, Waltham, MA, USA). As a reference for *CYP21A2,* the sequence NM_000500.9 was used, and the genetic variants detected in the target region were named according to human genome variation nomenclature (HGVS).

### 2.3. Statistical Analysis

All parameters were presented as medians with interquartile ranges for continuous variables or as counts and frequencies (%) for categorical variables. Chi-square and Fisher’s exact tests were used to investigate differences between categorical variables. After Kolmogorov–Smirnov and Shapiro–Wilk tests for normality of the distribution, non-parametric tests (Mann–Whitney U and Kruskal–Wallis tests) were used to explore the differences between groups. Additionally, after natural logarithm (ln) transformation of parameters, linear regression was used, adjusting for obesity and medication use.

A *p*-level < 0.05 was accepted as statistically significant. The Bonferroni adjustment for multiple testing was subsequently applied, and the significance of the *p*-value was set at <0.013 (0.05/4, considering the four investigated polymorphisms). The data were analyzed by MedCalc^®^ Statistical Software version 23.4.8 (MedCalc Software Ltd., Ostend, Belgium; https://www.medcalc.org; 2026) (accessed on 6 June 2026).

## 3. Results

A variety of *CYP21A2* nucleotide substitutions were detected in our study. The pathogenic variant *CYP21A2* c.844G>T; p.(Val282Leu) was found in 8 of the 12 patients with NC-CAH (66.7%): two of them were homozygous for this variant (confirmed by MLPA), five were compound heterozygous: c.844G>T; p.(Val282Leu) and another pathogenic allele (in three of them, variant c.293-13A/C>G), and one was a heterozygous carrier for c.844G>T; p.(Val282Leu) only. The genetic findings are shown in Table 1.

Conversely, none of the investigated 80 patients with PCOS carried the genetic substitution *CYP21A2* c.844G>T; p.(Val282Leu). Thus, the prevalence of pathogenic alleles among the genotyped 160 alleles was 0%, with 95% CI (0.0–2.3%) according to the exact binomial Clopper–Pearson method (Sergeant, ESG, 2018. Epitools Epidemiological Calculators. Ausvet. http://epitools.ausvet.com.au, retrieved on 6 June 2026).

Clinical, metabolic, and hormonal characteristics of the PCOS group are presented in Table 2.

**Figure 1:**
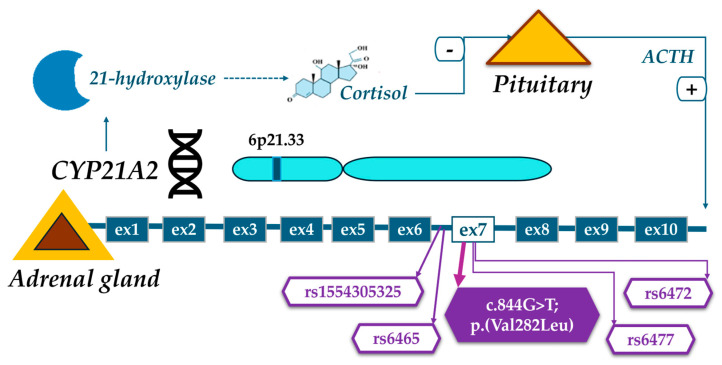
Genetic region and investigated polymorphisms in women with polycystic ovary syndrome. ACTH—adrenocorticotropic hormone. *CYP21A2*—21-hydroxylase gene.

Four polymorphic genetic variants were detected in the region of interest among women with PCOS (Figure 1); the allele distribution is presented in Table 3.

### 3.1. Polymorphism rs1554305325, c.738+12delinsGT in Women with PCOS (Old Nomenclature: c.735+12delinsGT)

The homozygous and heterozygous rs1554305325 polymorphic GT variant carriers were combined due to the small number of homozygotes. The presence of the GT variant was not related to the investigated clinical signs (acne, hirsutism, obesity, or menstrual irregularities) in women with PCOS (*p* > 0.05 for all).

Morning cortisol levels were higher in patients with polymorphic alleles than in those with wild-type alleles, and ACTH levels showed a similar trend (Table 4). Other metabolic and hormonal parameters did not differ between women with PCOS with different rs1554305325 genotypes (*p* > 0.05 for all).

### 3.2. Polymorphism rs6465, c.739-21C>T in Women with PCOS (Old Nomenclature: c.736-21C>G)

The rs6465 polymorphism was not associated with acne, hirsutism, obesity, or menstrual irregularities in the investigated women (*p* > 0.05 for all). No differences in most metabolic and hormonal parameters were observed between patients with different genotypes (*p* > 0.05 for all); however, pregnenolone and ACTH levels were lower in the polymorphic C/T group compared with wild C/C carriers (Table 4).

### 3.3. Polymorphism rs6472, c.806G>C; p.(Ser269Thr) in Women with PCOS (Old Nomenclature: c.803G>C/p.Ser268Thr)

The rs6472 polymorphic variant was not related to the development of acne, hirsutism, obesity, or menstrual irregularities in the women with PCOS (*p* > 0.05 for all). Most metabolic and hormonal parameters were similar between patients with different rs6472 genotypes (*p* > 0.05 for all). ACTH and morning cortisol levels were higher in patients with the polymorphic G/C and C/C genotypes than in women with the usual G/G genotype (Table 4).

### 3.4. Polymorphism rs6477, c.747C>G; p.(Leu249=) in Women with PCOS (Old Nomenclature: c.744C>G/p.Leu248=)

The rs6477 polymorphic variant was also not related to the development of acne, hirsutism, obesity, or menstrual irregularities in the women with PCOS (*p* > 0.05 for all). Most metabolic and hormonal parameters were similar between patients with different rs6477 genotypes (*p* > 0.05 for all), while ACTH levels were lower in women with the polymorphic C/G and G/G genotypes than in patients with the C/C genotype (Table 4).

After Bonferroni correction for multiple comparisons, only the association between the rs6472 variant and cortisol levels remained close to borderline significance. The effect size was small-to-medium (r = 0.27, 95% CI [0.05, 0.47]), explaining about 7% of variation.

## 4. Discussion

A wide range of genetic variations in the *CYP21A2* gene has been reported to date, including disease-causing mutations and polymorphisms. Our results show that 67% of female patients with NC-CAH in the current study are *CYP21A2* c.844G>T; p.(Val282Leu) homozygous or heterozygous carriers, with a c.844G>T; p.(Val282Leu) allele frequency of 41.7% (10/24). It was acknowledged previously that *CYP21A2* c.844G>T; p.(Val282Leu) variant frequency is ethnic-specific; for instance, its prevalence reached 74% in Sicilian and Ashkenazi Israeli cohorts, 54.1% in Argentinian, and 46% in Greek patients with non-classic CAH [15,16,17,18]. On the contrary, the same allele frequency is very low in Asian countries, e.g., China and Japan [19,20].

The c.844G>T; p.(Val282Leu) variant has been associated with a 50-80% reduction in normal 21-hydroxylase activity attributed to reduced hemoprotein content and conformational changes [21,22,23,24]. The moderate reduction in enzyme activity is usually associated with mild clinical symptoms caused solely by adrenal androgen excess; however, approximately one-third of patients with NC-CAH carrying the same variant show insufficient cortisol response to synthetic ACTH stimulation [25]. Additionally, severe forms of CAH in c.844G>T; p.(Val282Leu) homozygotes have rarely been described [13]. Nevertheless, the most common clinical complaints of patients with NC-CAH carrying the c.844G>T; p.(Val282Leu) variant include hirsutism, menstrual disturbances, and premature pubarche, and even asymptomatic individuals have been described [26,27].

On the other hand, heterozygous c.844G>T; p.(Val282Leu) female carriers from different ethnic groups might also present with pronounced hyperandrogenism and increased stimulated 17-OH progesterone levels [28,29]. These findings could be explained by the negative dominant effect of the mutant *CYP21A2* c.844G>T; p.(Val282Leu) allele on the normal allele in the heterozygous state, reducing the enzyme activity by about 30% [30]. Therefore, several studies have investigated the prevalence of the c.844G>T; p.(Val282Leu) variant among hyperandrogenic women. Neocleous et al. genotyped the *CYP21A2* gene in 205 female Cypriot patients with hirsutism, acne, or premature pubarche. The authors found 35 heterozygous carriers of *CYP21A2* c.844G>T; p.(Val282Leu) (17% of the investigated hyperandrogenic group) [28]. Among other ethnic groups, heterozygous c.844G>T; p.(Val282Leu) carriers were found in 6.0% of women with PCOS in Italy and 1.8–3.0% of those in the USA and Greece [31,32,33]. Additionally, 3.3% of Chinese patients with acne and hirsutism were carriers of the same pathogenic variant [34]. On the contrary, we did not find c.844G>T; p.(Val282Leu) variant among the 160 genotyped alleles of women with PCOS, which confirms the ethnic heterogeneity in the pathologic CAH allele distribution worldwide.

The role of *CYP21A2* polymorphisms in hyperandrogenic states and adrenal disorders beyond CAH is poorly investigated. Recently, a large Chinese study showed that the genetic intron polymorphism rs6465, located near exon 7, might influence the development of severe acne, with genotype TT being protective, especially in men [35]. The authors suggested that the intron variation might modulate 21-hydroxylase activity by affecting gene regulatory sequences [35]. In our cohort, the rare rs6465 TT genotype was not found; additionally, the frequency of acne and hirsutism did not differ between heterozygous CT and wild-type CC women. However, women with the CT genotype showed lower ACTH and pregnenolone levels in the unadjusted model, suggesting a possible influence of the rs6465 polymorphism on adrenal steroidogenesis; however, these differences were no longer significant after adjustment for multiple confounders. The rs6472 polymorphism (c.806G>C; p.(Ser269Thr)) might also be associated with adrenocortical hormonal fluctuations, considering that minor C-allele carriers showed significantly higher cortisol levels compared with other women with PCOS. Interestingly, the same polymorphism has been studied in Addison’s disease, and the C allele has been related to a lower risk of autoimmune adrenalitis [36]. The protective effect of the rs6472 minor C allele might be explained by the linkage with HLA protective variants [36] or by variant influence on cortisol secretion. However, in vitro functional assay has shown similar activity of p.S268T mutant compared with wild-type 21-hydroxylase protein [24]. On the other hand, functional clinical studies in patients with PCOS are currently lacking; thus, the role of the same polymorphism in vivo remains obscure. According to our results, the benign rs1554305325 polymorphism might also be associated with cortisol levels of the women with PCOS. A large study of healthy individuals and patients with non-functioning adrenal incidentalomas has shown that specific *CYP21A2* haplotypes, comprising different polymorphic alleles, can modulate cortisol and 17-hydroxyprogesterone levels after ACTH stimulation, as well as basic aldosterone levels [37]. Thus, the influence of various *CYP21A2* polymorphisms and haplotype groups on adrenal steroidogenesis in women with PCOS warrants further investigation across different ethnic groups.

The present study has an exploratory character, and several important limitations should be noted. For instance, the lack of a control group of healthy women and the small number of participants reduced statistical power. Moreover, radioimmunological methods were used instead of the recommended “gold standard” liquid chromatography with tandem mass spectrometry [38], and levels of stress in the cohort were not evaluated. The study was focused only on CYP21A2 exon 7, and functional enzyme analysis was not performed. Moreover, after Bonferroni correction for multiple comparisons, only the association between the rs6472 variant and cortisol levels tended to be significant. Nevertheless, the study shows possible associations between *CYP21A2* polymorphisms and cortisol secretion in women with PCOS. Thus, the findings need to be confirmed in larger studies that consider the influence of *CYP21A2* haplotypes on adrenal hormonal production in healthy women and patients with PCOS and non-classic CAH. The observed endocrine changes associated with the investigated *CYP21A2* polymorphisms did not influence the PCOS phenotype or specific symptoms, rendering their clinical significance questionable. However, symptoms and signs in women with PCOS undergo marked age-related changes associated with androgen decrease and metabolic deterioration [39]. Thus, only a longitudinal follow-up of patients with PCOS could estimate the clinical significance of possible associations between different *CYP21A2* benign polymorphisms and cortisol levels in basic and stress-related conditions.

In conclusion, the c.844G>T; p.(Val282Leu) variant is very common among Bulgarian patients with NC-CAH but it has not been found in our cohort of women with PCOS, unlike other studies. Thus, more scientific data should be gathered to establish the ethnic-specific distribution of NC-CAH-related alleles in patients with PCOS. Additionally, some *CYP21A2* exon 7 benign polymorphisms might be associated with cortisol levels in patients with PCOS. Further larger studies are necessary to reveal the possible influence of benign *CYP21A2* variants on adrenal steroidogenesis in women with PCOS and to explore the underlying pathophysiological mechanisms.

## Figures and Tables

**Table 1:** Genetic findings (pathogenic and benign variants) of the eight female patients who carried the c.844G>T; p.(Val282Leu) variant; all of them had a hormonally proven diagnosis of non-classic congenital adrenal hyperplasia per current guideline [7], though one of them turned out to be a heterozygous carrier. N-number.

N	CYP21A2 Allele 1	CYP21A2 Allele 2	rs1554305325	rs6465	rs6477	rs6472
**1**	c.844G>T;p.(Val282Leu)	c.293-13A/C>G	AC/GT	C/C	C/G	G/G
**2**	c.844G>T;p.(Val282Leu)	c.293-13A/C>G	AC/GT	C/C	C/G	G/G
**3**	c.844G>T;p.(Val282Leu)	c.293-13A/C>G	AC/GT	C/C	C/G	G/G
**4**	c.844G>T;p.(Val282Leu)	c.955C>T; p.(Gln319*)	AC/GT	C/C	C/G	G/G
**5**	c.844G>T;p.(Val282Leu)	c.1069C>T; p.(Arg357Trp)	AC/GT	C/C	C/G	G/G
**6**	c.844G>T;p.(Val282Leu)	c.844G>T;p.(Val282Leu)	GT/GT	C/C	G/G	G/G
**7**	c.844G>T;p.(Val282Leu)	c.844G>T;p.(Val282Leu)	GT/GT	C/C	G/G	G/G
**8**	c.844G>T;p.(Val282Leu)	-	AC/GT	C/C	G/G	G/G

**Table 2:** Clinical and laboratory characteristics of women with polycystic ovary syndrome (PCOS). Data presented as median (interquartile range) or percentage (n). ACTH—adrenocorticotropic hormone. LDL—low-density lipoprotein; HDL—high-density lipoprotein.

Parameter	Women with PCOS (n = 80)	Parameter	Women with PCOS (n = 80)
Age (years)	24.00 (21.00–27.00)	HDL-cholesterol (mmol/L)	1.29 (1.07–1.50)
BMI (kg/m^2^)	27.67 (23.05–35.09)	Triglycerides (mmol/L)	0.88 (0.61–1.18)
Hirsutism (%)	72.5% (58)	Testosterone (nmol/L)	1.66 (1.28–2.06)
Acne (%)	41.25% (33)	17-OH progesterone (nmol/L)	4.15 (3.05–5.85)
Glucose (mmol/L)	5.20 (4.97–5.61)	Pregnenolone (ng/mL)	2.90 (1.77–4.54)
Cholesterol (mmol/L)	4.48 (4.02–5.04)	ACTH (pmol/L)	4.15 (2.48–7.80)
LDL-cholesterol (mmol/L)	2.90 (2.44–3.60)	Cortisol (nmolL)	390.00 (280.00–521.00)

**Table 3:** Genotypes of *CYP21A2* exon 7 region polymorphisms observed among investigated patients with PCOS in the present study.

Genetic Variant		Major Allele Homozygote		Heterozygote		Minor Allele Homozygote	
		Geno-Type	N	Geno-Type	N	Geno-Type	N
rs1554305325	c.738+12delinsGT	AC/AC	57	AC/GT	19	GT/GT	4
rs6465	c.739-21C>T	C/C	73	C/T	7	-	0
rs6472	c.806G>C, p.(Ser269Thr)	G/G	55	G/C	21	C/C	4
rs6477	c.747C>G, p.(Leu249=)	C/C	55	C/G	22	G/G	3

**Table 4:** Laboratory characteristics of women with polycystic ovary syndrome according to *CYP21A2* exon 7 region polymorphisms. BMI—body mass index; DHEAS—dehydroepiandrosterone sulfate; ACTH—adrenocorticotropic hormone; 17OHP—17-hydroxy progesterone. Data are presented as median (interquartile range). *p*—Mann–Whitney test; *p* *—linear regression model adjusted for obesity (BMI ≥/<30), metformin and LT-4 use; similar results were obtained after exclusion of the two women on oral contraceptives in the last three months.

	rs6465				rs1554305325			
	C/C Genotype (n = 73)	C/T Genotype (n = 7)	*p*	*p* *	AC/AC Genotype (n = 57)	AC/GT or GT/GT Genotype (n = 23)	*p*	*p* *
Age (years)	24.0 (21.0–27.0)	26.0 (23.0–31.0)	0.203		25.0 (21.8–28.0)	22.0 (21.0–24.7)	0.127	
BMI (kg/m^2^)	27.8 (23.3–34.4)	27.3 (21.0–37.4)	0.845		28.0 (23.3–36.0)	27.6 (22.8–33.6)	0.632	
Metformin use	32.9% (24)	57.1% (4)	0.232		33.3% (19)	39.1% (9)	0.616	
L-thyroxin use	12.3% (9)	42.9% (3)	0.065		17.5% (10)	8.7% (2)	0.492	
Testosterone (nmol/L)	1.7 (1.3–2.1)	1.4 (1.3–1.6)	0.272	0.708	1.7 (1.3–2.1)	1.6 (1.3–2.1)	0.746	0.568
DHEAS (µmol/L)	9.27 (6.8–13.1)	7.27 (6.5-14.8)	0.536	0.923	8.7 (7.0–13.7)	10.4 (5.7–11.5)	0.887	0.456
17-OHP (nmol/L)	4.10 (2.9-5.8)	5.5 (3.6-6.4)	0.367	0.342	4.5 (3.0-5.9)	3.9 (3.0-4.8)	0.856	0.647
Pregnenolone (ng/mL)	3.1 (1.8–4.6)	1.43 (1.3 – 2.5)	**0.031**	0.077	2.9 (1.8–4.5)	3.1 (1.6–4.6)	0.920	0.994
Cortisol (nmol/L)	396.0 (283.0–521.5)	253.5 (186–380)	0.154	0.105	371.5 (232.0–445.5)	460.5 (372.0–530.0)	**0.038**	**0.035**
ACTH (pmol/L)	4.5 (2.9–8.3)	2.5 (2.3–3.2)	**0.030**	0.070	3.6 (2.4–6.7)	5.5 (3.1–9.7)	0.059	0.076
	rs6472				rs6477			
	**G/G Genotype** **(n = 55)**	**G/C or C/C** **Genotype (n = 25)**	***p***	p *	**C/C Genotype** **(n = 55)**	**C/G or G/G Genotype (n = 25)**	***p***	p *
Age (years)	25.0 (21.2–27.8)	23.0 (21.0–25.3)	0.269		23.0 (21.0–27.0)	25.0 (21.8–27.2)	0.428	
BMI (kg/m^2^)	28.0 (23.4–36.4)	27.6 (22.7–33.9)	0.513		28.0 (24.0–35.2)	25.7 (21.5–34.4)	0.237	
Metformin use	34.5% (19)	36.0% (9)	1.000		30.9% (17)	44.0% (11)	0.314	
L-thyroxin use	16.4% (9)	12.0% (3)	0.744		10.9% (6)	24.0% (6)	0.177	
Testosterone (nmol/L)	1.7 (1.3–2.1)	1.6 (1.3–2.2)	0.971	0.748	1.7 (1.3–2.2)	1.6 (1.3–2.0)	0.464	0.538
DHEAS (µmol/L)	9.1 (6.9–13.9)	9.8 (6.3–11.4)	0.732	0.602	9.1 (6.0–12.8)	10.4 (7.3–14.7)	0.455	0.326
17-OHP (nmol/L)	4.5 (3.1–5.9)	3.9 (3.0–5.2)	0.736	0.839	4.2 (3.0–6.0)	3.9 (3.0–5.5)	0.564	0.401
Pregnenolone (ng/mL)	2.7 (1.8–4.4)	3.2 (1.7–4.6)	0.804	0.888	3.1 (1.9–4.5)	2.4 (1.4–4.6)	0.161	0.202
Cortisol (nmol/L)	366.5 (232.0–442.0)	460.5 (373.0–546.0)	**0.016**	**0.013**	390 (281.0–493.0)	392.5 (240.5–565.5)	0.717	0.791
ACTH (pmol/L)	3.4 (2.4–6.5)	5.5 (3.1–9.9)	**0.022**	**0.020**	4.8 (2.9–8.5)	3.1 (2.3–5.2)	**0.048**	0.059

## Data Availability

Data are available after reasonable request.
